# Self-Organization of Fullerene Derivatives in Solutions and Biological Cells Studied by Pulsed Field Gradient NMR

**DOI:** 10.3390/ijms232113344

**Published:** 2022-11-01

**Authors:** Irina A. Avilova, Alexander V. Chernyak, Yuliya V. Soldatova, Alexander V. Mumyatov, Olga A. Kraevaya, Ekaterina A. Khakina, Pavel A. Troshin, Vitaliy I. Volkov

**Affiliations:** 1 Federal Research Center for Problems of Chemical Physics and Medicinal Chemistry RAS, 142432 Chernogolovka, Russia; 2 Scientific Center in Chernogolovka RAS, 142432 Chernogolovka, Russia; 3 A.N. Nesmeyanov Institute of Organoelement Compounds RAS, 119334 Moscow, Russia

**Keywords:** fullerene derivatives, associates, solution, red blood cells, pulsed field gradient NMR, self-diffusion, lifetime, lateral diffusion

## Abstract

Fullerene derivatives are of great interest in various fields of science and technology. Fullerene derivatives are known to have pronounced anticancer and antiviral activity. They have antibacterial properties. Their properties are largely determined by association processes. Understanding the nature and properties of associates in solvents of various types will make it possible to make significant progress in understanding the mechanisms of aggregation of molecules of fullerene derivatives in solutions. Thus, this work, aimed at studying the size and stability of associates, is relevant and promising for further research. The NMR method in a pulsed field gradient was used, which makes it possible to directly study the translational mobility of molecules. The sizes of individual molecules and associates were calculated based on the Stokes–Einstein model. The lifetime of associates was also estimated. The interaction of water-soluble C_60_ fullerene derivatives with erythrocytes was also evaluated. The values of self-diffusion coefficients and the lifetime of molecules of their compounds in cell membranes are obtained. It is concluded that the molecules of fullerene derivatives are fixed on the cell surface, and their forward movement is controlled by lateral diffusion.

## 1. Introduction

Fullerene is a three-dimensional allotropic modification of carbon (C*_n_*, where *n* = 20…540). A fullerene molecule is a convex closed polyhedron consisting of an even number of atoms forming pentagons and hexagons. Currently, a large number of types of fullerenes are known, but the most common and studied is the C_60_ molecule. At the vertices of a truncated icosahedron are 60 carbon atoms, and the faces are 20 hexagons and 12 pentagons. Fullerene derivatives are widely used in various fields of science and technology [1,2].

Water-soluble fullerene derivatives (WSFD) show considerable promise in the field of biomedical applications. These are substances with unique properties. They can be used as a targeted component of drug delivery systems. It is known from the literature data that some of the fullerene derivatives exhibited pronounced anticancer activity [3,4,5,6], antiviral (including anti-HIV) [7,8,9,10,11], and antibacterial [4,12,13] activity. It was also shown that fullerenes and fullerene-dye hybrid structures have a pronounced antitumor photodynamic effect [14,15,16].

The biological activity of water-soluble fullerene derivatives is explained by the peculiarity of their structure. Water-soluble fullerene derivatives are amphiphilic compounds consisting of a hydrophobic carbon framework surrounded by hydrophilic addends [17,18,19]. As a result, these compounds are lipophilic. On the one hand, WSFDs interact with the lipid matrix of the cell membrane; on the other hand, WSFDs interact specifically with protein active sites.

There are many publications devoted to the behavior of WSFD in proteins, model and native biological membranes [20,21,22,23,24]. However, at the same time, only a few works are devoted to studying the interaction of water-soluble fullerene derivatives with red blood cell (RBC) membranes [25,26]. Although RBCs as a biological system are of interest since the structure of their membrane is similar to the membranes of other cells. In [25,26], fullerenols C_60_(OH)_24_ and C_60_(OH)_36_ containing polar hydroxyl groups were studied. The authors showed that fullerenols are predominantly associated with surface proteins of the plasma membrane; however, they can also be incorporated into the membrane. It has also been demonstrated that fullerenol affects membrane ATPases and can modulate ion transport across membranes.

The amphiphilic nature of the molecules of water-soluble fullerene derivatives causes their aggregation in aqueous solutions. As a result of self-organization processes, nanosized hollow spherical vesicles are formed [27,28,29]. It is reasonable to assume that the biological properties of these molecules will be influenced by the processes of self-aggregation and the size of the resulting clusters. Therefore, the study of self-organization in solutions of fullerene derivatives is of fundamental importance.

The processes of self-organization of fullerene derivatives in water and organic solutions are studied using various experimental methods: dynamic light scattering [7,30,31,32], atomic force microscopy [7,30,33,34], transmission electron microscopy [7,32,33,35,36]. However, these methods are rather indirect.

The most direct way to estimate the size of aggregates in solutions from diffusion coefficients is using the Stokes–Einstein model. Diffusion NMR spectroscopy is informative for measuring molecular diffusion parameters [37,38]. For example, in [39,40,41,42], the ^1^H DOSY NMR method was used to study supramolecular systems based on fullerenes. This method made it possible to obtain information about the processes of formation of supramolecular systems by analyzing the self-diffusion coefficients of the initial compounds and finished complexes. It was shown that the mobility of supramolecular systems is lower than the mobility of the initial molecules. In paper [43], the ^1^H DOSY NMR method was used to establish the formation of fullerene polyesters, which was also confirmed by a decrease in the diffusion coefficient.

A special place in the field of diffusion NMR spectroscopy is occupied by the pulsed field gradient NMR technique (PFG NMR). In the PFG NMR experiment, individual diffusion components could be selected from the NMR spectra using Fourier transform, and the partial diffusion coefficients can be estimated, which makes it possible to apply this method to study the association of fullerene derivatives in organic and aqueous media.

Another remarkable feature of PFG NMR is the possibility of its application to study the translational mobility of molecules in biological systems (cells, models) [22,23,44,45,46,47,48,49,50,51,52,53]. It does not have a destructive effect on cells and, at the same time, allows obtaining of qualitative and quantitative information related to the self-diffusion processes of molecules in biosystems.

Thus, the following conclusions can be drawn. First, water-soluble fullerene derivatives are promising compounds for biomedical research and pharmaceutical applications. Second, the investigation of these substances’ state in aqueous solutions, as well as their interaction with biological systems (for example, RBCs), is very important. Third, the pulsed field gradient NMR technique makes it possible to noninvasively study the translational mobility of fullerene derivatives molecules in solutions and biological cells.

Herein we have presented the experimental results of fullerene derivative association in solutions and erythrocytes obtained with the pulsed field gradient technique.

## 2. Results and Discussion

### 2.1. Fullerene Derivatives in Solutions

The Stokes–Einstein model is usually used to describe the aggregation of molecules in solutions. The hydrodynamic radius *r_H_* of a spherical particle is calculated using the self-diffusion coefficient *D* according to Equation (1) [54,55,56,57,58]:

(1) $$D=\frac{k\cdot T}{n\cdot \pi \cdot \eta \cdot r_{H}},$$

where *k* is the Boltzmann constant, *T* is the absolute temperature, and *η* is the solvent viscosity.

In the case of slip molecules, the value of *n* is equal to four. For the stick-shaped molecules, *n* is equal to 6.

The applicability of the Stokes–Einstein model for calculating the size of particles formed by molecules of fullerene derivatives was tested using the fluorinated fullerene C_60_F_36_. The structure of this compound is well known; the van der Waals diameter is 1.23 nm [58]. From the experimental data of PFG NMR of ^19^F nuclei, the value of the self-diffusion coefficient of C_60_F_36_ molecules was obtained, which was 6.5 10^−10^ m^2^/s [59]. The hydrodynamic diameter 2*r_H_*, calculated from Equation (1), is equal to the van der Waals diameter at *n* = 6 within the measurement error. Thus, it seems appropriate to apply Equation (2) for hydrodynamic radii of particles formed by molecules of fullerene derivatives estimation:

(2) $$r_{H}=\frac{k\cdot T}{6\cdot \pi \cdot \eta \cdot D}$$

#### 2.1.1. Nonpolar Fullerene Derivatives

**Figure 1:**
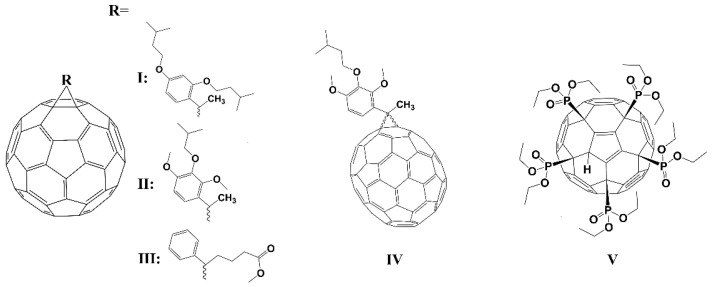
The molecular structure of compounds **I**–**V**.

The molecular structures of fullerene derivatives that do not contain polar groups (**I**–**V**) are shown in Figure 1.

For compounds **I**–**V**, spin echo attenuation of ^1^H nuclei was measured in the solvents indicated in Section 3.1. The dependences of the spin echo attenuation amplitude *A*(2*τ*, *τ*_1_, *g*) vs. the square of the pulsed field gradient amplitude *g*^2^ (diffusion decays) were analyzed. The diffusion decays were exponential shapes and fitted well using Equation (3):

(3) $$A\left(2\tau ,\tau_{1},g\right)=A\left(2\tau ,\tau_{1},0\right)\exp\left(-\gamma^{2}g^{2}\delta^{2}t_{d}D_{s}\right),$$

which is an instance of Equation (9) presented in Section 3.3 at *m* = 1.

The exponential character of diffusion decays of compounds **I**–**V** indicates that the mobility of the molecules of fullerene derivatives is characterized by a single self-diffusion coefficient *D_s_*. Since the NMR spectra of the studied compounds are well resolved, the diffusion decays of different spectral lines belonging to different functional groups were analyzed. The resulting decays turned out to be almost identical.

Table 1 presents the self-diffusion coefficients obtained for the molecules of compounds **I**–**V** in various solvents. The sizes of particles *d_H_* = 2·*r_H_* are calculated using the Stokes–Einstein Equation (2).

The hydrodynamic diameters of the molecules of compounds **I**–**V** are in the range of 1.2–1.4 nm. It should be noted that the type of solvent does not affect the particle sizes, which are close to the van der Waals diameter of the fullerene. Thus, fullerene derivatives containing non-polar groups are not prone to the formation of aggregates but are in solutions as isolated molecules.

#### 2.1.2. Polar Fullerene Derivatives

**Figure 2:**
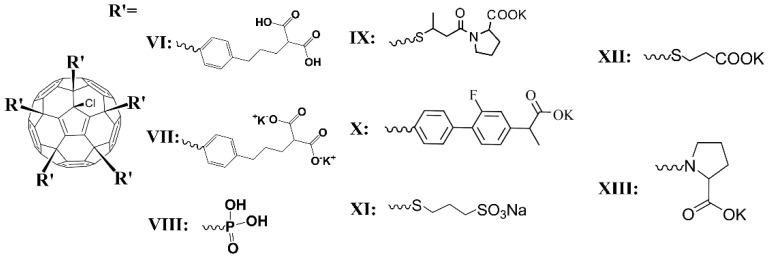
The molecular structure of compounds **VI**–**XIII**.

Another picture was observed for fullerene derivatives containing polar groups. The molecular structures of fullerene derivatives containing polar groups are shown in Figure 2.

The exponential diffusion decays were observed for solutions of **VI** in deuterated DMSO-d_6_, acetone-d6, and DMSO-D_2_O mixtures. The fullerene self-diffusion coefficients and hydrodynamic diameters calculated from Equations (2) and (3), respectively, are shown in Table 2.

The calculated particle size formed by the molecules of compound **VI** in solutions of deuterated DMSO and acetone was 2.3–2.4 nm. The obtained value exceeds the size of the C60 fullerene molecule twice as much. Presumably, it can be explained by the presence of five bulk addends on the C_60_ fullerene framework, which increases the molecular hydrodynamic diameter. Another option is the formation of self-assembling dimers by compound **VI** due to the occurrence of hydrogen bonds between the -COOH groups of two complementary molecules, as is well known for organic acids.

At the same time, in a mixture of DMSO-D_2_O solvents, for the molecules of compound **VI**, a noticeable aggregation was observed, which can be seen in Table 2. Probably, stable aggregates of various sizes, including D_2_O molecules, are formed in the system. In addition, the observed exponential diffusion decay characterizes an average aggregate self-diffusion coefficient.

**Figure 3:**
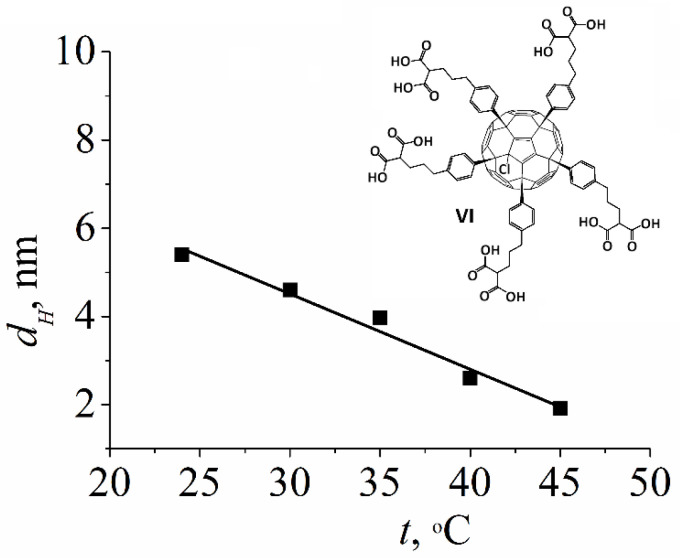
Temperature dependence of the hydrodynamic diameter *d_H_* characterizing the association of the fullerene derivative **VI** in DMSO/D_2_O (3:1 m/m) solution. The concentration of **VI** was 8 mg/mL.

Associates formed by the molecules of compound **VI** possess low stability, which was confirmed using the dependence of the hydrodynamic diameter *d_H_* on temperature. Figure 3 shows that with the increasing temperature of the solution from 25 to 45 °C, a decrease in particle size was observed.

#### 2.1.3. Water-Soluble Fullerene Derivatives

The results obtained for compounds **VII**–**XIII** in aqueous solutions should be considered separately.

##### Compound **VII**

**Figure 4:**
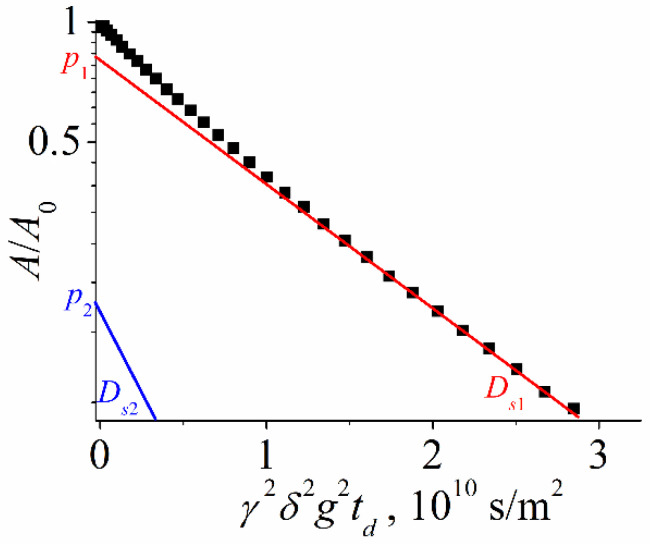
Diffusion decay of the fullerene derivative **VII** in D_2_O solution at *t_d_* = 20 ms, *C* = 6 mg/mL.

Water-soluble fullerene derivatives (e.g., compound **VII** in Figure 2) demonstrate the strongest tendency to self-organization in solutions. The self-diffusion of the potassium salt **VII** in D_2_O was investigated. The diffusion decays were approximated by the sum of two exponential components according to Equation (4) [59] (*m* is equal to two in Equation (9)) as it is shown in Figure 4:

(4) $$A\left(g\right)=p_{1}\exp\left(-\gamma^{2}g^{2}\delta^{2}t_{d}D_{s1}\right)+p_{2}\exp\left(-\gamma^{2}g^{2}\delta^{2}t_{d}D_{s2}\right),$$

where *p*_1_, *D_s_*_1_ and *p*_2_, *D_s_*_2_—are the relative parts (phase populations) and partial self-diffusion coefficients of the first and the second component, respectively.

The hydrodynamic diameters *d_H_* based on each self-diffusion coefficient were calculated from Equation (2) and are given in Table 3.

From the data in Table 3, we can conclude that there are two different types of clusters formed by the fullerene derivative in the solution. Based on the obtained values of hydrodynamic diameters, it can be assumed that isolated molecules (*d_H_* = 2 nm) and stable aggregates (*d_H_* about 5.5 nm) are observed. The values of the self-diffusion coefficients did not depend on the solution concentration; they were constant in the concentration range from 6 to 24 mg/mL. The increasing of aggregate populations with concentration rising was observed.

##### Compound **VIII**

The results obtained for the fullerene derivative **VIII** show a completely different behavior of molecules in the solution. It is assumed that for the molecules of this compound, the aggregate lifetime is shorter than the diffusion time. This ensures fast exchange between isolated molecules and aggregates of compound **VIII**. Therefore, the obtained diffusion decays are exponential. Their analysis revealed average self-diffusion coefficients characterizing the translational mobility of various types of compound **VIII** aggregates.

**Figure 5:**
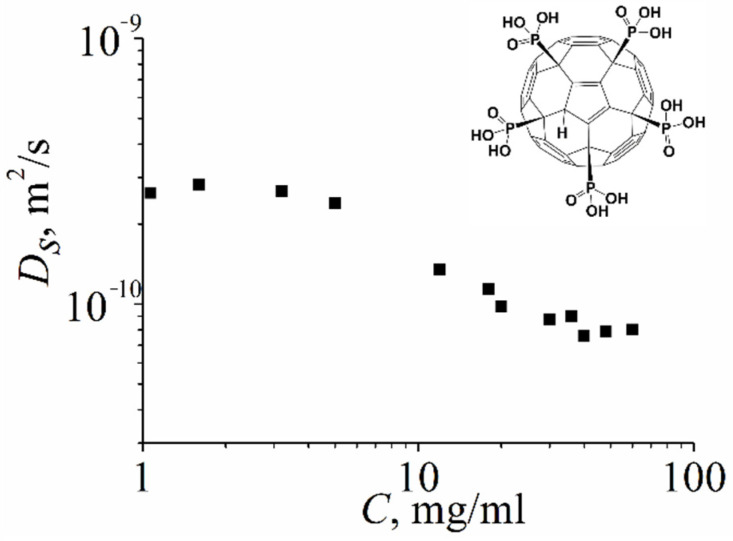
The concentration dependence of self-diffusion coefficients of compound **VIII** in D_2_O measured on ^31^P nuclei.

Figure 5 shows the dependence of the self-diffusion coefficient of molecules of compound **VIII** on the concentration of an aqueous solution. At low solution concentrations (less than 3 mg/mL), the *d_H_* value was (1.3 ± 0.15) nm, which corresponds to the size of an isolated molecule. With an increase in concentration, a decrease in the self-diffusion coefficient was observed, which indicates the formation of aggregates by the molecules of the fullerene derivative **VIII**. At a solution concentration of 40 mg/mL, the maximum value of the hydrodynamic diameter *d_H_* = (4.5 ± 0.5) nm was obtained, which does not change with a further increase in concentration.

##### Compound **IX**

**Figure 6:**
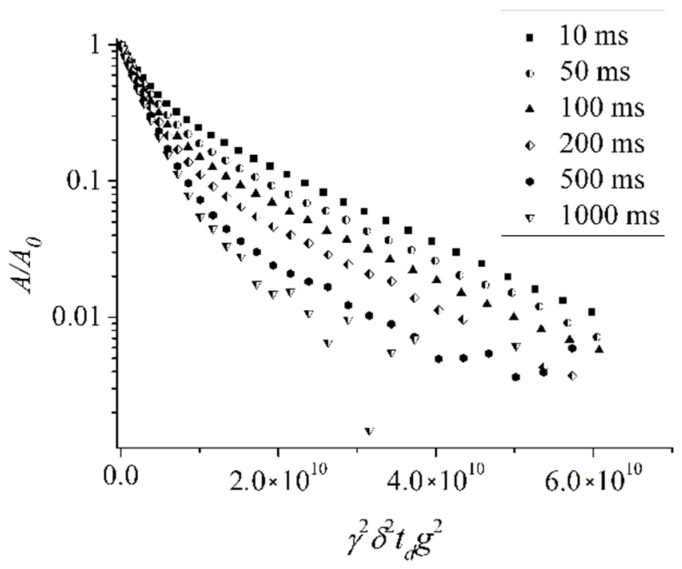
Diffusion decays of the fullerene derivative **IX** at different diffusion times *t_d_*. The values of *t_d_* are indicated in insertion. *C* = 64.4 mg/ml. Reprinted with permission from Ref. [37]. Copyright © 2016 Published by Elsevier B.V.

For compound **IX**, aqueous solutions of the concentrations indicated in paragraph 4.1 were prepared, and NMR experiments were performed. The diffusion decays of fullerenes (Figure 6) were approximated using bi-exponential curves according to Equation (4).

From the analysis of diffusion decays, the self-diffusion coefficients of the molecules of compound **IX** were obtained, the values of which were *Ds*_1_ = (6.5 ± 0.3) 10^−11^ m^2^/s and *Ds*_2_ = (4.0 ± 0.5) 10^−10^ m^2^/s.

By analogy with the previous compounds, it is assumed that *Ds*_1_ and *Ds*_2_ characterize particles of the fullerene derivative of different sizes. From Equation (2), the particle sizes were calculated, the values of which were *d_H_*_1_ = (5.2 ± 0.4) nm and *d_H_*_2_ = (1.0 ± 0.06) nm. The obtained values indicate that the molecules of compound **IX** in an aqueous solution are represented by isolated and aggregated molecules.

The experimental data obtained for compound **IX** give the possibility to estimate the lifetime of aggregates formed by molecules in an aqueous solution. The lifetime of fullerene aggregates was estimated from the temperature dependence of the low self-diffusion coefficient population on diffusion time *t_d_ p*_2_(*t_d_*) according to the procedure proposed in [37].

**Figure 7:**
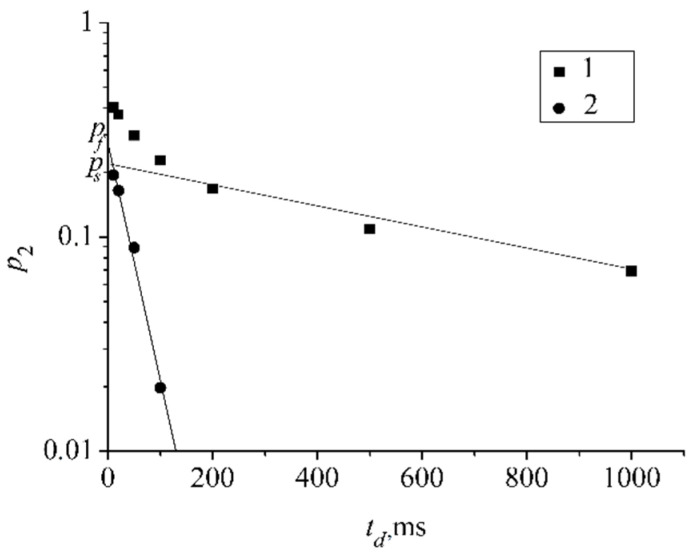
1—the dependence of part *p*_2_(*t_d_*) on diffusion time *t_d_*; 2—the dependence *p*_2_(*t_d_*) after subtraction of spin–lattice relaxation component. Reprinted with permission from Ref. [37]. Copyright © 2016 Published by Elsevier B.V.

Figure 7 shows an example of dependence *p*_2_(*t_d_*). The characteristic times of the curve tail were about 1 s, which was in good agreement with the values of the spin lattice relaxation times (*T*_1_ = 0.9 s) for this compound, measured independently. Figure 7 also shows the result of the spin lattice relaxation component subtracting, which is a straight line. Their slopes were used to calculate the lifetime τ according to Equation (5):

(5) $$p_{2}=p_{f}\exp\left(-\frac{t_{d}}{\tau }\right)+p_{s}\exp\left(-\frac{t_{d}}{T_{1}}\right),$$

where *τ* is the lifetime, *T*_1_ is fullerene spin lattice relaxation time, *p_f_* + *p_s_* = *p*_2_(0).

The lifetime values *τ* are 140 ms, 180 ms, and 200 ms for compound **IX** solution concentrations of 64.4 mg/mL, 21.3 mg/mL, and 5.3 mg/mL, respectively.

##### Compound **X**

**Figure 8:**
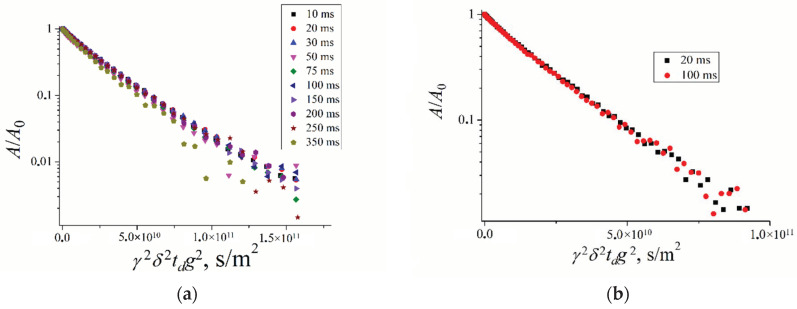
10 Diffusion decays of ^1^H and ^19^F nuclei of compound **X** at different diffusion times *t_d_*: (**a**)—^1^H nuclei; (**b**)–^19^F nuclei. The values of *t_d_* show in insertions. The aqueous solution concentration is 50 mg/mL.

For compound **X**, aqueous solutions of the concentrations indicated in Section 3.1 were prepared. NMR studies were performed for ^1^H and ^19^F nuclei. The diffusion decays of ^1^H (Figure 8a) and ^19^F (Figure 8b) nuclei at different diffusion times *t_d_* are shown.

These diffusion decays are bi-exponential shapes. As shown in Figure 8, self-diffusion coefficients *D_s_*_1_ and *D_s_*_2_ are *t_d_* independent. It may be proposed that two types of molecule associations are observed. The exchange rate between these two associates was slow compared to diffusion time. Self-diffusion coefficients decrease with solution concentration increasing. Hydrodynamic diameters calculated from Stokes–Einstein by Equation (2) are given in Table 4. The lesser diameter is close to individual molecule diameters, but the largest diameter characterizes associate size.

The particle sizes calculated from ^1^H and ^19^F self-diffusion data are agreed well. The associate size, as well as the relative part of associated molecules, increases with the solution concentration increasing. The diameter increases from 7.6 nm to 9.8 nm and from 7.6 nm to 10.6 nm; the associate part increases from 0.67 to 0.8 and from 0.69 to 0.83 for ^1^H and ^19^F data, correspondingly, with concentration increasing from 6 to 50 mg/mL.

##### Compound **XI**

For compound **XI**, aqueous solutions of the concentrations indicated in Section 3.1 were prepared. NMR studies were performed on ^1^H nuclei. These derivative diffusion decays are bi exponential which was evidence of two sizes of particles: individual molecules *d_H_*_2_ = (0.9 ± 0.1) nm and associated molecules *d_H_*_1_ = (5.6 ± 0.3) nm.

**Figure 9:**
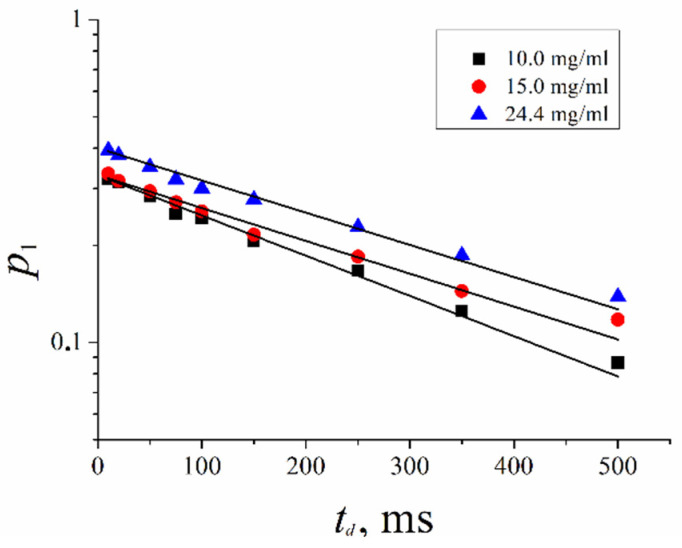
Dependences of associate molecules populations of compound **XI** on diffusion times. The values of concentrations are indicated in insertion.

As shown in Figure 9, the low diffusion associated molecule component population *p*_1_ decreases with diffusion time *t_d_* increasing. In Figure 9, *p*_1_(*t_d_*) dependence is shown.

This dependence is exponential, as described by Equation (6). The lifetime of associates formed by molecules of compound **XI** in aqueous solutions was calculated.

(6) $$p\left(t_{d}\right)=p\left(0\right)\exp\left(-\frac{t_{d}}{\tau }\right)$$

The lifetime of associates *τ* are (350 ± 10), (430 ± 20) and (440 ± 20) ms at solution concentration *C* = 10, 15 and 24.4 mg/mL, accordingly. The lifetime of associates increased insignificantly with solution concentration increasing.

##### Compound **XII**

The diffusion decay of compound **XII** is bi-exponential, which is due to isolated (self-diffusion coefficient *D_s_*_2_ = (4.3 ± 0.8)·10^−10^ m^2^/s) and associated molecules (self-diffusion coefficient *D_s_*_1_ = (7.5 ± 1.5)·10^−11^ m^2^/s). The dependence of diffusion decay shape on diffusion time *t_d_* and concentration was negligible, indicating a slow exchange between isolated and associated molecules compared to the maximum value of *t_d_* (250 ms). The isolated molecule size *d_H_*_2_ = 1.0 ± 0.2 nm, and the associate size was *d_H_*_1_ = 5.0 ± 1.0 nm. The relative part of associates increases with concentration growth from 0.56 to 0.73.

##### Compound **XIII**

The ^1^H diffusion decay for molecules of compound **XIII** is also bi-exponential, and decay shape depends on diffusion time and solution concentration *C* insignificantly. Self-diffusion coefficients *Ds*_1_ and *Ds*_2_ are (1.3 ± 0.1)·10^−10^ m^2^/s and (5.8 ± 0.2)·10^−10^ m^2^/s, accordingly. Hydrodynamical diameters are *d_H_*_2_ = (0.7 ± 0.1) nm—isolated molecules and *d_H_*_1_ = (2.8 ± 0.2) nm—associates. The associated molecules’ relative part increases from 0.59 to 0.70, with concentration increasing from 3 to 40 mg/mL.

### 2.2. Water-Soluble Fullerene Derivatives in Red Blood Cells

Water-soluble fullerene derivatives (WSFDs) are biologically active species. Therefore, one of the fundamental problems is to reveal the mechanism of fullerene derivative interaction with Red Blood Cells (RBCs).

**Figure 10:**
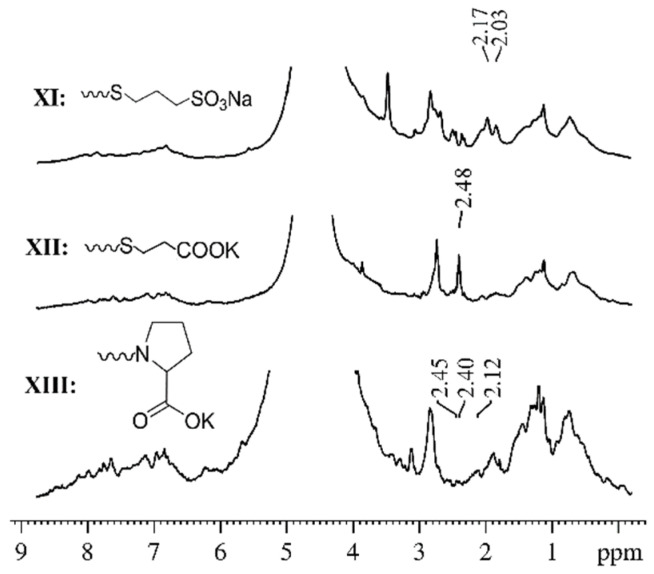
The ^1^H spectra of the RBCs suspensions with added compounds **XI**–**XIII**.

We have previously performed a detailed study of the translational mobility of compound **XI** in various biological systems: liposomes, shadows, and erythrocytes [53]. In this work, analysis of the high-resolution NMR spectra of the following systems was conducted: an aqueous solution of compound **XI**; RBCs suspension; suspensions of RBCs, RBCs shadows, and liposomes with the addition of compound **XI**. Using the analysis of ^1^H spectra at different amplitudes of the magnetic field gradient, we showed that the signals from the protons of compound **XI** could be uniquely identified in the spectra of suspensions. These signals do not overlap with the proton signals of the membrane components. It was also shown that, at a high amplitude of the magnetic field gradient, in the spectra of ^1^H suspension of RBCs with added compound **XI**, a signal of WSFD molecules was well observed. At the same time, proton signals of compound **XI** molecules are absent in an aqueous solution under the same experimental conditions. A similar picture was obtained for compounds **XII** and **XIII**. Figure 10 shows examples of the ^1^H spectra of the systems under study (erythrocyte suspensions with added compounds **XI**–**XIII**). ^1^H chemical shifts of compounds are slightly changing in suspensions of RBCs compared to solutions due to fullerene derivative interaction with RBCs.

The isolated ^1^H signals of compound molecules give the possibility to register the diffusion decays of WSFD molecules in a suspension of RBCs correctly. The obtained experimental data made it possible to extract information about the translational mobility of WSFD molecules in cell suspensions. The obtained diffusion decays were complex and were decomposed into three exponent components according to Equation (9) at *m* = 3. At the same time, in aqueous solutions of compounds **XI**–**XIII**, two exponential diffusion decays are observed. The values of the self-diffusion coefficients of the molecules of compounds **XI**–**XIII** are presented in Table 5 [44].

As shown in Table 5, the translational mobility of molecules of compounds **XI**–**XIII** in a suspension of RBCs is characterized by three diffusion coefficients, the values of which differ by an order of magnitude. However, only two self-diffusion coefficients have been identified in the aqueous solutions of these compounds. Extraction of the third coefficient of self-diffusion, the value of which differs significantly from the other two coefficients, suggests that the movement of the molecules of compounds **XI**–**XIII** in the cell suspension was hindered.

It is known from the literature data that the lateral diffusion coefficient *D_L_*, which characterizes the mobility of lipid molecules in the cell membrane, has a value of about (5–7)∙10^−12^ m^2^/s [44,52,53]. The observed “low” coefficient of self-diffusion of the molecules of compounds **XI**–**XIII** was close to a lateral diffusion coefficient. Thus, the complex nature of the obtained experimental data can be explained by the fact that in the erythrocyte suspension, the molecules of compounds **XI**–**XIII** are in the aqueous phase of the system or are bound to the erythrocyte cell membrane. The penetration of molecules of compounds **XI**–**XIII** into the membrane was observed as a result of the appearance of a slow diffusion component. At the same time, in the aqueous phase, the molecules of compounds **XI**–**XIII** are in an isolated and associated form.

The analysis of the dependence of relative parts slow moving fullerene derivative molecules *p*_1_ on diffusion time *t_d_* makes it possible to estimate the lifetimes *τ* of fullerene derivative molecules in the erythrocyte membrane from Equation (6). Lifetimes *τ* for compounds **XI**–**XIII** are given in Table 6.

**Figure 11:**
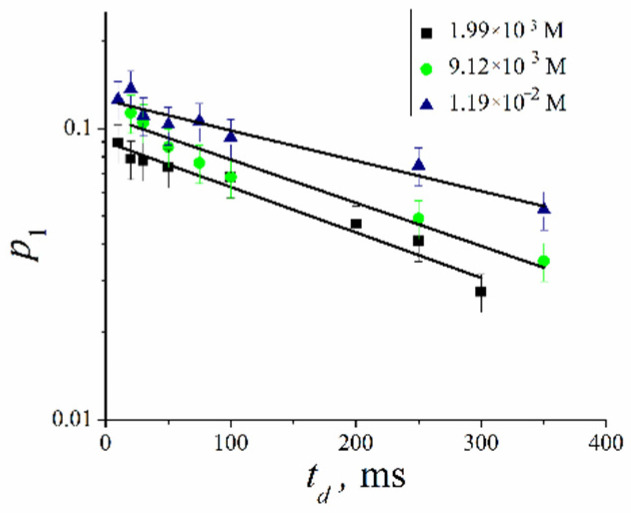
The dependences of the relative parts *p*_1_ of molecules of compound **XI** on the diffusion time *t*_d_ at various concentrations. The concentrations indicate in the insertion.

The dependences of the relative parts *p*_1_ of molecules of compound **XI** on the diffusion time *t*_d_ at different concentrations are shown in Figure 11. From the analysis of these dependences, the lifetime calculation was carried out according to Equation (6). The obtained values of the lifetimes are presented in Table 7.

As seen from Table 7, the lifetime *τ*_1_ of compound **XI** molecules in the cell membrane increases with the increase of its concentration in the RBCs suspension.

## 3. Materials and Methods

### 3.1. Fullerene Derivatives

Methanofullerenes **I**, **II,** and **IV** were reported recently [60,61]. Compound **III** represents a well-known n-type material for organic solar cells abbreviated as PCBM [62]. Fullerene derivatives **V** and **VIII** were synthesized recently in the reaction of chlorofullerene C_60_Cl_6_ and triethyl phosphite [17]. Fullerene derivatives **VI** and **VII** were reported as products of the Friedel–Crafts arylation of C_60_Cl_6_ [63]. Compounds **IX and XI** were synthesized as described in [64]. Fullerene derivatives **X**, **XII**, and **XIII** were obtained and characterized as reported in our previous papers [19], [65], [66], respectively.

The molecular mobility of compounds **I**–**V** was studied in various solvents: CDCl_3_ (**I**, **II**, **III**, **V**), CS_2_ (**I**, **II**), and C_6_D_5_CD_3_ (**II**, **IV**). For investigation of the molecular mobility of compound **VI** in solution, deuterated DMSO-d6, acetone-d6, and mixtures of DMSO-D_2_O were used. Compounds **VII**–**XIII** are highly soluble in water due to the presence of five hydrophilic groups. Therefore, solutions of various concentrations in deuterium water were prepared for them. The concentrations were from 6 to 24 mg/mL for **VII**; from 2 to 50 mg/mL for **VIII**; from 5 to 64 mg/mL for **IX**; from 6 to 50 mg/mL for **X**; from 10 to 24 mg/mL for **XI**; from 10 to 47 mg/mL for **XII**; from 3 to 40 mg/mL for **XIII**.

### 3.2. Red Blood Cells

Blood samples were taken from anesthetized mice by decapitation. The 0.11 molar sodium citrate aqueous solutions were used as an anticoagulant. The citrate solution to blood ratio was one to five by volume. The collected blood samples were centrifuged for 15 min at 1000 rpm, and the plasma was removed with decantation. The erythrocyte sediment was resuspended in NaCl solution (salt concentration was 0.85 g per 100 mL) containing 5 mM of Na-phosphate buffer with pH = 7.4. The centrifugation and decantation procedures were repeated triply; the duration of the second and third centrifugations was 7 min. The fresh buffered NaCl solution was used for each resuspending step. The obtained erythrocyte sediment was stored at 4 °C for no more than 36 h. The fullerene derivatives were added to the RBCs suspension. The final concentration of WSFD in suspension was 1.19·10^−2^, 9.12·10^−3^, and 1.99·10^−3^ M for compound **XI**; 1.2·10^−2^ M for compound **XII** and **XIII**.

### 3.3. PFG NMR Technique

**Figure 12:**
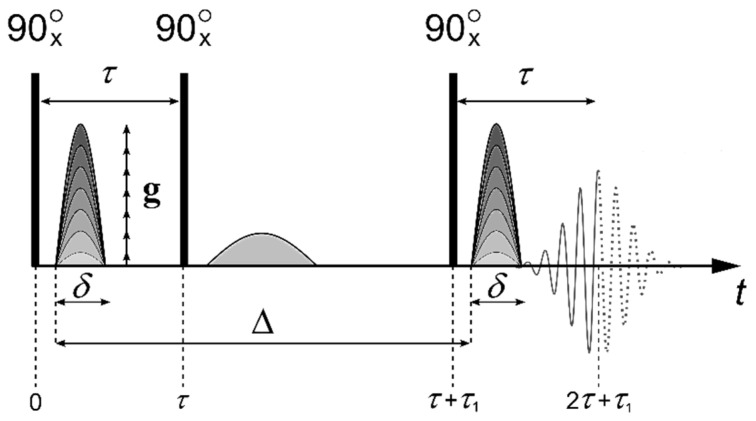
Stimulated echo pulse sequence with the magnetic field gradient pulses. Here, *τ* is the time interval between the first and the second RF pulses, and *τ*_1_ is the time interval between the second and the third ones. *D* is the interval between the gradient pulses, *δ* is the duration of the equivalent rectangular magnetic field gradient pulses, *g* is the amplitude of the magnetic field gradient pulse, and *g*_0_ is the amplitude of the constant magnetic field gradient. Reprinted with permission from Ref. [44].

The self-diffusion coefficients were measured with the pulsed field gradient technique for ^1^H, ^19^F, and ^31^P nuclei at the frequencies of 400.13, 376.498, and 161.976 MHz, correspondingly. The measurements were conducted on Bruker Avance-III—400 NMR spectrometer equipped with the diff-60 gradient unit. The pulsed field gradient stimulated echo sequence shown in Figure 12 was applied in the experiments. Three 90° pulses produce a stimulated spin-echo at the time of 2*τ* + *τ*_1_ (where *τ* and *τ*_1_ are the time intervals between the first and the second or the second and the third 90° pulses, respectively). The magnetic field gradient pulses of amplitude *g* and duration *δ* were applied after the first and the third 90° pulses. The gradient strength was varied linearly in 64 steps within the range from 0.1 to 27 T/m. The integrated intensities of the spectrum lines were used to obtain the dependence of the echo signal attenuation with respect to *g*^2^ (diffusion decay) [44,49,50,51,52,67].

For the molecules undergoing unhindered isotropic Brownian motion, the evolution of the spin echo signal is described by the following Equation (3):

(3) $$A\left(2\tau ,\tau_{1},g\right)=A\left(2\tau ,\tau_{1},0\right)\exp\left(-\gamma^{2}g^{2}\delta^{2}t_{d}D_{s}\right),$$

where *γ* is gyromagnetic ratio, *t_d_* = Δ−*δ*/3 is the diffusion time, and *D_s_* is the self-diffusion coefficient, *τ*, *τ*_1_ and *g* are shown in Figure 12; *A*(2*τ*, *τ*_1_, 0) is expressed by the equation:

(7) $$\left(2\tau ,\tau_{1},0\right)=\frac{A\left(0\right)}{2\exp\left(-\frac{2\tau }{T_{2}}-\frac{\tau_{1}}{T_{1}}\right)}$$

where *A*(0) is the signal intensity after the first radio frequency (RF) pulse (Figure 12). *T*_1_ and *T*_2_ are the spin-lattice and spin-spin relaxation times, respectively. While measuring the echo signal evolution, *τ* and *τ*_1_ are fixed, and only the dependence of *A* as a function of *g* is analyzed, which is called the diffusion decay.

In the case of non-exponential diffusion decay decays, the experimental curves:

(8) $$A\left(g\right)=\frac{A\left(2\tau ,\tau_{1},g\right)}{A\left(2\tau ,\tau_{1},0\right)},$$

are usually deconvoluted in several exponential components, which are described by Equation (3). For the multiphase system consisting of *m* phases in the case of slow (compared to *t_d_*) molecular exchange between the phases:

(9) $$A\left(g\right)=\overset{m}{\underset{i=1}{\sum }}p_{i}^{\prime }\exp(-\gamma^{2}g^{2}\delta^{2}t_{d}D_{si})$$

where *D_si_* is the self-diffusion coefficient of the *i*-th component and:

(10) $$p_{i}^{\prime }=p_{i}\exp\left(-\frac{2\tau }{T_{2i}}-\frac{\tau_{1}}{T_{1i}}\right)/\overset{m}{\underset{i=1}{\sum }}p_{i}\exp\left(-\frac{2\tau }{T_{2i}}-\frac{\tau_{i}}{T_{1i}}\right),\;\overset{m}{\underset{i=1}{\sum }}p_{i}=1$$

Here *p_i_* is the relative amount of the nuclei belonging to the molecules characterized by the self-diffusion coefficient *D_si_.* The value *p_i_* is called the population of the *i*-th phase. For the long *T*_1_ and *T*_2_ values, it is usually assumed that *p_i_* ≈ *p*′*_i_*. The details of the experimental curve decomposition in several exponential diffusion decays were described previously [44].

We have decomposed diffusion decays on two or three exponential components; the values *m* in Equation (9) were two or three, accordingly. Between different phases (individual and associated molecules), a molecular exchange occurs. The lifetime in phase is *τ*. In the case of fast exchange rate *τ << t_d_* (*t_d_* is diffusion time), the exponential diffusion decay is observed, which is characterized by the average self-diffusion coefficient:

*D* = Ʃ*p_i_*∙*D_si_* (11)

For slow rate exchange conditions, *τ >> t_d_* multi-exponential diffusion decay shape according to Equation (9) is observed, and *p_i_* and *D_si_* do not depend on diffusion time *t_d_*.

If phase exchange time is comparable with diffusion time *τ* ≈ *t_d_*, the slowly moving molecule phase population exponentially decreases with increasing diffusion time, and lifetime *τ* may be estimated [49,50].

## 4. Conclusions

The self-organization of a series of differently functionalized fullerene derivatives dissolved in the solvents of different polarity and suspension of RBCs was investigated with pulsed field gradient NMR spectroscopy of ^1^H, ^19^F, ^31^P nuclei. No aggregation was observed for the fullerene derivatives containing nonpolar functional groups **I**–**V** in carbon disulfide, deuterated chloroform, and toluene-*d*_8_ solutions. The hydrodynamic diameters calculated from Stokes–Einstein equation are equal to individual molecule van der Waals diameters (1.2–1.4 nm). Therefore, Stokes–Einstein model is correct for hydrodynamic size calculations. The fullerene derivatives bearing polar functional groups such as -COOH, -COOK, -P(O)(OH)_2_, -SO_3_Na showed a strong tendency to aggregate. The revealed diameters of the aggregates varied from 2.2 nm to 9.6 nm depending on the solvent and temperature. The water-soluble fullerene derivatives **VII**–**XIII** demonstrated the most stable aggregates with a diameter of about 5 nm. An analysis of the concentration dependences showed that in solutions, the number of associated molecules of water-soluble derivatives of fullerenes increases with increasing concentration. Aggregated lifetimes were estimated from the analysis of diffusion decay shape dependence on the diffusion time. Associate lifetime increases with increasing fullerene derivatives aqueous solution concentration.

Self-diffusion of water-soluble fullerene derivatives **XI**–**XIII** in RBCs was characterized using ^1^H PFG NMR. Were obtained and analyzed the diffusion decays of the molecules of compounds **XI**–**XIII** in a suspension of RBCs. It was found that a fraction of fullerene derivative molecules shows a self-diffusion coefficient of about (5–6)·10^−12^ m^2^/s, independent of fullerene derivative type, which matched the coefficient of the lateral diffusion of lipids in the RBCs membrane (*D_L_* = (5–7) 10^−12^ m^2^/s). This experimental finding evidences the absorption of the fullerene derivatives by RBC. The obtained results suggest that fullerene derivative molecules are probably fixed on the RBC surface. The average lifetime of the fullerene derivative molecule on RBC was estimated as 440 ± 70 ms for compound **XI**, 470 ± 70 ms for compound **XII**, and 1200 ± 300 ms for compound **XIII**. An experimental dependence of the lifetime of molecules of compound **XI** in the RBC membrane on the concentration of the compound in suspension was obtained. It was shown that with increasing concentration, the lifetime also increases.

Thus, pulsed field gradient NMR was shown to be a versatile technique for the investigation of self-organization and interactions of the fullerene derivatives with blood cells, providing essential information which could be projected on their behavior in-vivo after intravenous administration while screening as potential drug candidates.

## Figures and Tables

**Table 1:** The self-diffusion coefficients *D_s_* and the diameters *d_H_* calculated from Equation (2) for the fullerene derivatives **I**–**V**.

Compound	Solvent	*D_s_*, m^2^/s	*d_H_*, nm
**I**	CDCl_3_	5.9·10^−10^	1.3 ± 0.1
	CS_2_	1.0·10^−9^	1.2 ± 0.1
**II**	CDCl_3_	6.3·10^−10^	1.3 ± 0.1
	CS_2_	1.1·10^−9^	1.2 ± 0.1
	C_6_D_5_CD_3_	6.6·10^−10^	1.2 ± 0.1
**III**	CDCl_3_	6.9·10^−10^	1.2 ± 0.1
**IV**	C_6_D_5_CD_3_	6.1·10^−10^	1.2 ± 0.1
**V**	CDCl_3_	5.8·10^−10^	1.4 ± 0.1

**Table 2:** The self-diffusion coefficients *D_s_* and the diameters *d_H_* from Equation (2) for fullerene derivative **VI**, the solution concentration *C* = 8 mg/mL.

Solvent	*D_s_*, m^2^/s	*d_H_*, nm
acetone-d6	5.7·10^−10^	2.4 ± 0.2
DMSO	8.5·10^−11^	2.3 ± 0.2
The molar ratio DMSO:D_2_O = 3:1	2.1·10^−11^	5.4 ± 0.5
The molar ratio DMSO:D_2_O = 1:1	2.3·10^−11^	6.3 ± 0.6
The molar ratio DMSO:D_2_O = 1:3	2.3·10^−11^	9.6 ± 0.9

**Table 3:** The self-diffusion coefficients *D_s_*_1_, *D_s_*_2_, populations *p*_1_, *p*_2_, and the hydrodynamic diameters *d_H_*_1_, *d_H_*_2_ of the aggregates formed by the compound **VII** in deuterated water solutions.

*C*, mg/mL	*D_s_*, m^2^/s		*p*		*d_H_*, nm	
	*D_s_*_1_	*D_s_*_2_	*p*_1_	*p*_2_	*d_H_*_1_	*d_H_*_2_
6	7.1·10^−11^	2.1·10^−10^	0.60	0.40	5.5 ± 0.5	1.8 ± 0.2
12	6.6·10^−11^	1.7·10^−10^	0.70	0.30	5.9 ± 0.6	2.3 ± 0.2
14	7.1·10^−11^	1.7·10^−10^	0.80	0.20	5.5 ± 0.5	2.3 ± 0.2
24	6.9·10^−11^	2.0·10^−10^	0.87	0.13	5.7 ± 0.6	1.9 ± 0.2

**Table 4:** Diameters of particles of compound **X** calculated on the basis of Equation (2) from self-diffusion coefficients measured for ^1^H and ^19^F nuclei.

*C*, mg/mL	^1^H		^19^F	
	*d_H_*_1_, nm	*d_H_*_2_, nm	*d_H_*_1_, nm	*d_H_*_2_, nm
6	7.6 ± 0.4	0.8 ± 0.1	7.6 ± 0.4	1.0 ± 0.1
8	7.6 ± 0.4	1.0 ± 0.1	7.8 ± 0.4	1.0 ± 0.1
12	7.4 ± 0.4	1.1 ± 0.1	8.2 ± 0.4	1.1 ± 0.1
16	7.6 ± 0.4	1.1 ± 0.1	8.0 ± 0.4	1.1 ± 0.1
24	8.0 ± 0.4	1.3 ± 0.1	8.4 ± 0.4	1.6 ± 0.1
30	7.9 ± 0.4	1.5 ± 0.1	8.8 ± 0.4	1.9 ± 0.1
50	9.8 ± 0.4	2.4 ± 0.1	10.6 ± 0.4	2.4 ± 0.1

**Table 5:** Self-diffusion coefficients of WSFD molecules in aqueous solutions and RBCs suspension. *D_s_*_1_^w^ and *D_s_*_2_^w^—aqueous solution**;** *D_s_*_1_^s^, *D_s_*_2_^s^*, D_s_*_3_^s^—RBCs suspension. Reprinted with permission from Ref. [44].

Compound	Aqueous Solution		RBCs Suspension		
	*D_s_*_1_^w^·10^11^, m^2^/s	*D_s_*_2_^w^·10^10^, m^2^/s	*D_s_*_1_^s^·10^12^, m^2^/s	*D_s_*_2_^s^·10^11^, m^2^/s	*D_s_*_3_^s^·10^10^, m^2^/s
**XI**	7.4 ± 0.7	4.1 ± 0.4	5.5 ± 0.8	3.9 ± 0.6	5.5 ± 0.8
**XII**	7.5 ± 1.5	4.3 ± 0.8	5.0 ± 1.0	4.4 ± 0.9	7.1 ± 1.4
**XII**	4.9 ± 0.5	1.2 ± 0.1	6.0 ± 1.0	3.8 ± 0.6	8.0 ± 1.0

**Table 6:** The self-diffusion coefficients *D_s_*_1_^s^, population *p*_1_ at *t_d_*→0*, p*_1_(0), and lifetimes *τ* of WSFD molecules in RBCs. Reprinted with permission from Ref. [44].

Compound	*D_s_*_1_^s^·10^12^, m^2^/s	*p*_1_(0)	*τ*, ms
**XI**	5.5 ± 0.8	0.33	440 ± 70
**XII**	5.0 ± 1.0	0.13	470 ± 70
**XIII**	6.0 ± 1.0	0.06	1200 ± 300

**Table 7:** The values of the lifetime of the molecules of compound **XI** in the RBC membrane at various concentrations.

*C*, M	*τ*_1,_ ms
1.99·10^−3^	280 ± 15
9.12·10^−3^	300 ± 20
1.19·10^−2^	440 ± 70
